# Paternal cholestasis exacerbates obesity-associated hypertension in male offspring but is prevented by paternal ursodeoxycholic acid treatment

**DOI:** 10.1038/s41366-018-0095-0

**Published:** 2018-05-24

**Authors:** Vanessa Pataia, Georgia Papacleovoulou, Vanya Nikolova, Anne-Maj Samuelsson, Stephanie Chambers, Eugene Jansen, Paul D Taylor, Lucilla Poston, Catherine Williamson

**Affiliations:** 1 Department of Women and Children’s Health, King’s College London, London, SE1 7EH UK; 2 Institute of Reproductive and Developmental Biology, Imperial College London, London, W12 0NN UK; 3 Centre for Health Protection, National Institute for Public Health and the Environment, PO Box 1, Bilthoven, 3720 BA The Netherlands

**Keywords:** Cardiovascular diseases, Metabolic diseases

## Abstract

**Background:**

Obesity is a heterogeneous phenotype and risk associations to non-communicable diseases such as cardiovascular disease and type 2 diabetes are influenced by several factors. The paternal metabolic status at the time of conception influences offspring susceptibility to developing obesity and adiposity-associated cardiometabolic disease. Cholestatic liver diseases are characterized by raised circulating serum bile acid levels and dyslipidemia, and are commonly treated with ursodeoxycholic acid (UDCA). We hypothesized that paternal cholestasis alters offspring susceptibility to developing obesity and adiposity-associated cardiometabolic disease and that this may be modified by paternal UDCA treatment.

**Methods:**

Cholestasis was induced in male C57BL/6 mice with a 0.5% cholic acid (CA)-supplemented diet for 10 weeks prior to mating with normal chow (NC)-fed females. Offspring of cholestatic and NC-fed fathers were fed either a NC diet or challenged with an obesogenic ‘western diet’ (WD) from 12 weeks of age. Offspring body weight and cardiometabolic function were assessed, and the impact of treatment of paternal cholestasis with UDCA was evaluated.

**Results:**

Male offspring (18 weeks old) of cholestatic fathers challenged with WD had raised fasting insulin, hepatic triglyceride content and serum cholesterol levels compared to diet-matched controls. At 25–29 weeks of age, WD-fed male offspring of cholestatic fathers had higher systolic and diastolic blood pressure than controls and this was prevented by paternal UDCA treatment. In contrast, WD-challenged female offspring of cholestatic fathers showed improved glucose tolerance compared to controls.

**Conclusions:**

We demonstrated in our model of paternal cholestasis that offspring susceptibility to adiposity-associated cardiometabolic disease is affected in a sex-specific manner and paternal UDCA treatment had a protective effect against hypertension in the obese male offspring. The most prevalent human cholestatic conditions are primary sclerosing cholangitis and primary biliary cholangitis. These findings are of clinical relevance to children of men with these conditions.

## Introduction

Currently, there is a world-wide epidemic of non-communicable diseases (NCDs) such as cardiovascular disease and type 2 diabetes. Obesity is one of the main risk factors for cardiovascular disease and type 2 diabetes [1]. However, there is no linear relationship between body mass and NCDs and additional factors may influence individual disease risk [2].

Accumulating evidence suggests that not only maternal health, but also the paternal metabolic status at the time of conception can impact the subsequent health of the offspring [3]. For example, paternal obesity has been associated with long-term alterations in body fat percentage of prepubertal daughters [4] and early-onset paternal obesity is associated with raised alanine aminotransaminase in the offspring [5]. Food availability of grandfathers also impacts upon longevity and cardiovascular risk in the progeny, as reported in studies of a Swedish population [6].

Epigenetic marks in sperm may provide a mechanistic pathway through which offspring disease susceptibility can be altered, a hypothesis supported by studies in rodent models of paternal obesity, diabetes and manipulation of dietary folate [7–9]. Changes in sperm DNA methylation, histone modifications and small RNA content have also been associated with increased disease susceptibility in offspring of fathers exposed to diet-induced obesity [9–13], low-protein diet [14] or stress [15, 16]. Additionally, the paternal seminal plasma has been shown to influence the female oviduct cytokine expression profile and impact the offspring phenotype [17].

Cholestatic liver conditions are characterized by an impairment of bile acid efflux from the liver resulting in the accumulation of bile acids in the circulation concomitant with dyslipidemia [18]. The most common chronic cholestatic liver diseases affecting men are primary sclerosing cholangitis and primary biliary cholangitis with a prevalence of up to 16.2 per 100,000 persons and 40.2 per 100,000 persons respectively in the general population [19].

We have previously reported that offspring exposed to maternal cholestasis during gestation are predisposed to developing increased adiposity, abnormal lipid profiles, diabetes and non-alcoholic fatty liver disease [20]. In addition, a previous study has shown that 4 months of 0.5% cholic acid (CA) dietary supplementation to male mice resulted in reduced fertility, concomitant with decreased sperm count and smaller offspring litters. Further analysis showed that after 2 months of CA diet, the blood–testis barrier was no longer intact and cell aggregates were visible in the center of the seminiferous tubules and this was associated with decreased CX43 accumulation in gap junctions [21].

In this study, we used a mouse model to establish whether paternal cholestasis influences offspring susceptibility to developing obesity and adiposity-associated cardiometabolic disease. As cholestatic conditions are typically treated with ursodeoxycholic acid (UDCA) [18], we have also explored the effect of paternal cholestasis treatment with UDCA on offspring health.

## Materials and methods

### Animal experiments

All procedures were approved by the Animal Welfare and Ethical Review Body at King’s College London and carried out according to the UK Animals (Scientific Procedures) Act 1986. Male and female C57BL/6 mice aged 6–8 weeks were purchased from Harlan Laboratories, UK. Mice were housed on a 12 h:12 h light/dark cycle with ad libitum access to food and water. Male mice were assigned to either a RM3 normal chow (NC) diet (*n* = 6) or a RM3 diet supplemented with 0.5% CA (*n* = 10) (LBS Serving Biotechnology, UK). Male mice were kept on the assigned diet for 10 weeks and body weight and food intake was measured weekly. Male mice fed a NC or CA diet were mated to NC-fed female mice of established fertility. Female mice used in the experiments had been previously mated and allowed to deliver 1 litter to establish fertility. During the mating period males were permanently kept with females and had access to ad libitum NC diet. Mating was confirmed by the presence of a copulatory plug, after which male mice were fasted for 4 h after 9 a.m. and killed by CO_2_ inhalation. Serum, liver, gonadal white adipose tissue, subcutaneous white adipose tissue and testes were weighed and snap-frozen. Females were allowed to give birth and litters were left undisturbed for 24 h. On day 2, pup number per litter and pup weight were assessed and litters were standardized to 5–6 pups. Offspring body weight was measured weekly thereafter. Pups were kept on a NC diet from 3 to 12 weeks of age, at which point half of the male and female offspring from each litter were challenged with a calorie-rich western diet (WD) (LBS Serving Biotechnology, UK) until 18 weeks of age. The combination of paternal and offspring exposure resulted in 4 experimental offspring groups: NC NC, CA NC, NC WD and CA WD (first two letters: paternal dietary exposure; second two letters: offspring dietary exposure) (Supplementary Figure 1). At 18 weeks of age, one male offspring (NC NC: *n* = 6, CA NC: *n* = 8, NC WD: *n* = 6, CA WD: *n* = 8) and one female offspring (NC NC: *n* = 4, CA NC: *n* = 8, NC WD: *n* = 5, CA WD: *n* = 10) per litter were killed by CO_2_ inhalation after 4 h of fasting from 9 a.m. and tissues harvested as described above. In a second cohort, the paternal feeding experimental protocol was repeated and a group of male mice fed a 0.5% cholic acid+0.5% ursodeoxycholic acid (CA+UDCA)-supplemented diet (LBS Serving Biotechnology, UK) was included (NC: *n* = 10, CA: *n* = 12, CA+UDCA: *n* = 9). Male offspring were fed a NC diet until 12 weeks of age and then transferred to WD. The 3 offspring groups according to paternal and offspring diet were NC WD, CA WD, CA+UDCA WD (Supplementary Figure 2). Cardiovascular recordings were made in male offspring (25–29 weeks) by radiotelemetry (NC: *n* = 4, CA: *n* = 3, CA+UDCA: *n* = 4) (see below).

### Glucose tolerance test (GTT)

At 18 weeks of age, one male (NC NC: *n* = 6, CA NC: *n* = 8, NC WD: *n* = 6, CA WD: *n* = 8) and one female offspring (NC NC: *n* = 4, CA NC: *n* = 8, NC WD: *n* = 4, CA WD: *n* = 8) per litter were fasted for 6 h from 9 a.m. followed by an intraperitoneal injection of 2 g/kg of d-glucose (Sigma-Aldrich, UK). Blood glucose was measured using a glucometer (AccuCheck, UK) before glucose injection and 15, 30, 45, 60 and 120 min following glucose injection. Tail blood samples were collected prior to glucose injection and 30 min after glucose injection for serum insulin measurements. Area under the curve (AUC) was calculated from the measurements from each mouse from before the start of GTT until 120 min following glucose injection.

### Radiotelemetry

Systolic blood pressure, diastolic blood pressure, heart rate and activity were measured in 1 male offspring per litter after surgical implantation of probes (Data Sciences International, USA) in the carotid artery (see supplementary information). Measurements are represented over a 24 h period in zeitgeber time where ZT 0 to ZT 12 is lights-on in the animal colony and ZT 12 to ZT 24 (ZT 0) is lights-off. Light and dark cycle averages were calculated based on the hourly averages taken between ZT 0 and ZT 11 and ZT 12 and ZT 24, respectively.

### Insulin measurements

Measurements of serum insulin from blood samples taken before the start of GTT and 30 min after the start of GTT were undertaken using the Mercodia Mouse Insulin enzyme-linked immunosorbent assay (ELISA; Mercodia, Sweden) according to the manufacturer’s protocol.

### Lipid measurements

Lipids were extracted from frozen liver samples using a lysis buffer containing 0.125 M potassium phosphate. Serum and lipids were run on a Unicel DxC 800 autoanalyzer (Beckman-Coulter, The Netherlands) for measurements of total cholesterol, low-density lipoprotein (LDL)-cholesterol, high-density lipoprotein (HDL)-cholesterol, triglycerides (TGs) and free fatty acids as previously described [22].

### Total RNA extraction and cDNA synthesis

Total RNA was extracted from frozen tissue samples using the RNeasy Mini kit (Qiagen, UK) according to the manufacturer’s instructions. For complementary DNA (cDNA) synthesis, total RNA was reverse transcribed using SuperScript™ II Reverse Transcriptase (Thermo Fisher Scientific, UK). RNase inhibition was used to prevent RNA digestion.

### Quantitative real time-PCR

The expression of target genes of interest was assessed using quantitative real time-PCR (RT-PCR) with a ViiA™ 7 Real Time PCR System (Thermo Fisher Scientific, UK). cDNA was added in duplicate followed by a reaction mix containing 1× of SYBR Green Jumpstart Readymix (Sigma-Aldrich, UK) and 1 μM of forward/reverse primers. The housekeeping gene *Cyclophilin b* was used as an internal reference for cDNA quality and relative quantification of gene expression. The genes assessed included *ATP-binding* cassette sub-family G member 5 and 8 (*Abcg5* and *Abcg8*), acetyl-CoA carboxylase 1 and 2 (*Acc1* and *Acc2*), bile salt export pump (*Bsep*), cholesterol 7-alpha-monooxygenase (*Cyp7a1*), sterol 12-alpha-hydroxylase (*Cyp8b1*), fatty acid synthase (*Fas*), HMG-CoA reductase (*Hmgcr*), stearoyl-CoA desaturase (*Scd1*), small heterodimer partner (*Shp*) and sterol regulatory element-binding protein 1c (*Srebp-1c*). Primer sequence list is provided in Supplementary Table 1.

### Statistical analysis

Data are presented as mean ± SEM. Statistical analysis was performed using GraphPad Prism 7 software (GraphPad Software Inc., USA). Data were checked for normality using the Shapiro–Wilk normality test. Repeated measures one-way analysis of variance (ANOVA) followed by Newman–Keuls post hoc test or two-way ANOVA followed by Tukey's post hoc test were applied for multiple comparisons. For single comparisons, unpaired two-tailed *t*-test was used. The significance cut-off was *P* ≤ 0.05.

## Results

### Cholestasis lowers paternal body weight and causes dyslipidemia

**Fig. 1:**
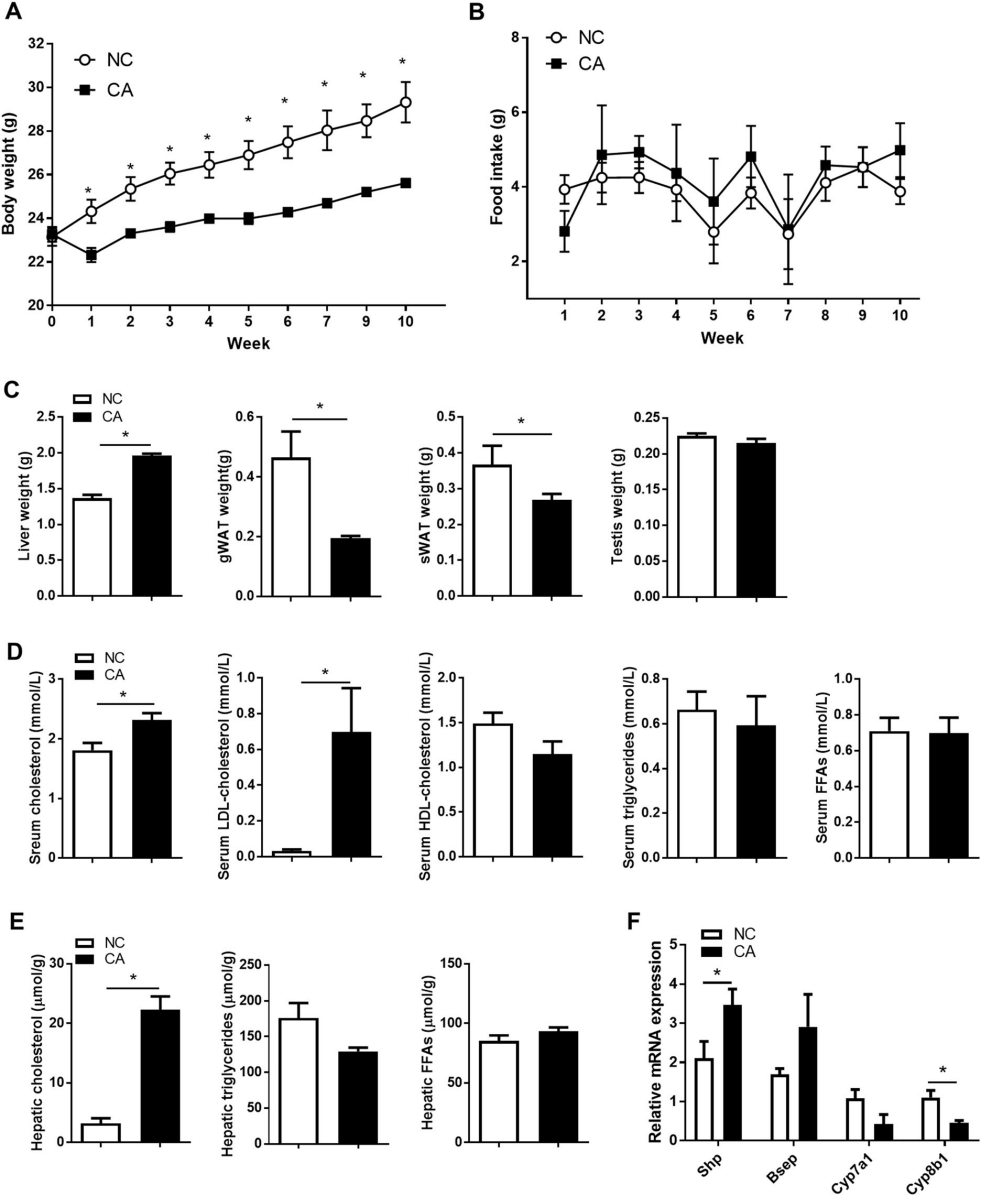
Paternal morphometry and metabolic profile. **a** Paternal body weight during feeding period; *n* = 6–10. **b** Paternal daily food intake during feeding period; *n* = 6–10. **c** Paternal organ weight; *n* = 6–10. **d** Paternal serum lipid levels; *n* = 6. **e** Paternal hepatic lipid levels; *n* = 6. **f** Paternal hepatic expression of bile acid homeostasis genes; *n* = 6. Data are presented as mean ± SEM. *P ≤ 0.05. Unpaired two-tailed *t*-test was used

We first established the effect of cholestasis induced by 10 weeks of 0.5% CA feeding on the paternal phenotype. CA-fed fathers were lighter than controls over the duration of the feeding period (Fig. 1a) and this was not explained by lower daily food intake (Fig. 1b). After 10 weeks of CA feeding, liver size was increased by 44% in cholestatic males, whereas gonadal white adipose tissue (gWAT) and subcutaneous white adipose tissue (sWAT) weight decreased by 58 and 18% respectively (Fig. 1c). No changes were observed in testis weight (Fig. 1c). Time to mate was not different between fathers from NC and CA groups (4.89 ± 1.4 vs 3.33 ± 0.67 days respectively) (Supplementary Figure 3).

CA feeding caused an aberrant paternal lipid profile, with raised serum total cholesterol and LDL-cholesterol levels (Fig. 1d) and increased hepatic cholesterol content (Fig. 1e). Serum and hepatic TG and free fatty acid (FFA) concentrations were not altered by CA feeding (Fig. 1d, e).

Investigation of the hepatic expression of key genes regulating bile acid homeostasis showed a pro-cholestatic profile with increased hepatic expression of the farnesoid x receptor (FXR) target *Shp* and reduced expression of *Cyp8b1* as compared to controls (Fig. 1f). Moreover, trends for increased expression of *Bsep* and decreased expression of *Cyp7a1* were observed.

### Offspring litter size, birth weight and morphometry

**Fig. 2:**
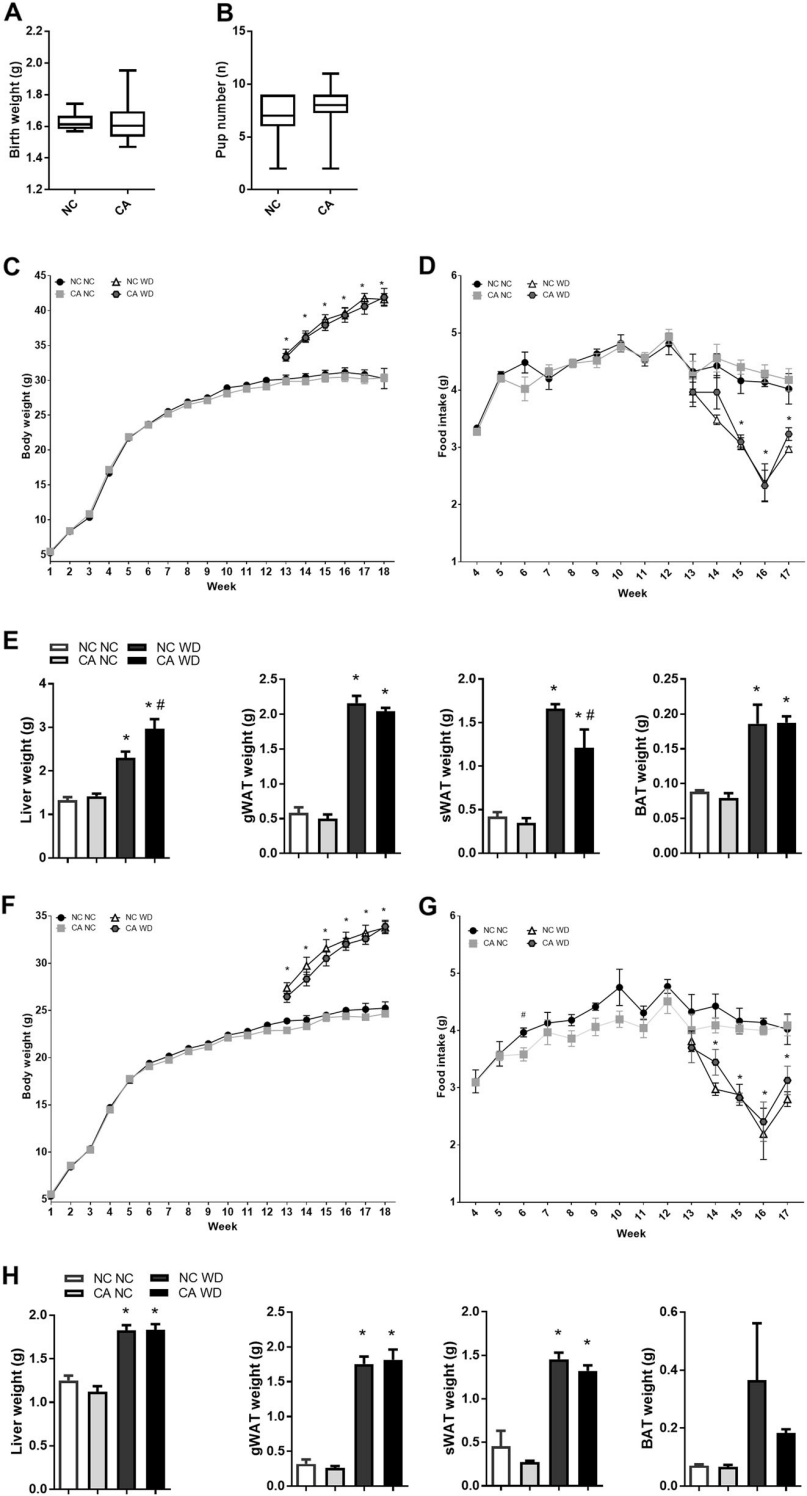
Offspring birth parameters and morphometry later in life. **a** Offspring birth weight; *n* = 6–10. **b** Offspring pup number per litter; *n* = 6–10. **c** Male offspring body weight from 1 to 18 weeks of age; *n* = 6–10. **d** Male offspring mean average daily food intake from 4 to 17 weeks of age; *n* = 6–10. **e** Male offspring organ weight; *n* = 6–8. **f** Female offspring body weight from 1 to 18 weeks of age; *n* = 4–10. **g** Female offspring mean daily food intake from 4 to 17 weeks of age; *n* = 4–10. **h** Female offspring organ weight; *n* = 4–10. Data are presented as mean ± SEM. *P ≤ 0.05 for effects due to WD challenge in comparisons vs NC NC and CA NC; ^#^*P* ≤ 0.05 for effects due to paternal cholestasis in comparisons of NC NC vs CA NC or NC WD vs CA WD. Repeated measures one-way ANOVA followed by Newman–Keuls *post hoc* test was used

Birthweight and number of pups per litter was not altered by paternal cholestasis (Fig. 2a, b).

Challenge with a WD resulted in an overall increase in male and female offspring body weight, as well as liver, gWAT, sWAT and brown adipose tissue weight in males (Fig. 2c, e, f, h). A decrease in food intake in WD-fed males and females as compared to NC-fed offspring was also observed (Fig. 2d, g).

Despite no differences in total body weight or food intake when compared to NC WD controls (Fig. 2c, d), 18-week-old WD-fed male offspring of cholestatic fathers (CA WD) showed a 29% increase in liver weight and a 26% decrease in sWAT weight (Fig. 2e). These changes were not observed when male offspring were maintained on a NC diet.

Female offspring of cholestatic fathers fed a NC or WD did not show significant changes in body weight when compared to female progeny of control fathers fed a matched diet (Fig. 2f). However, at 6 weeks of age female offspring of CA fathers (CA NC) had decreased daily food intake, a trend which persisted until 16 weeks of age (Fig. 2g). No differences in organ weight were observed between female offspring of cholestatic and control fathers when fed matched diets (Fig. 2h).

Overall, these results suggest that challenge with a calorie-rich diet affects male and female offspring of cholestatic fathers differently, with males being more susceptible to changes in organ morphometry.

### Paternal cholestasis has distinct effects on glucose homeostatic responses to western diet in male and female offspring

**Fig. 3:**
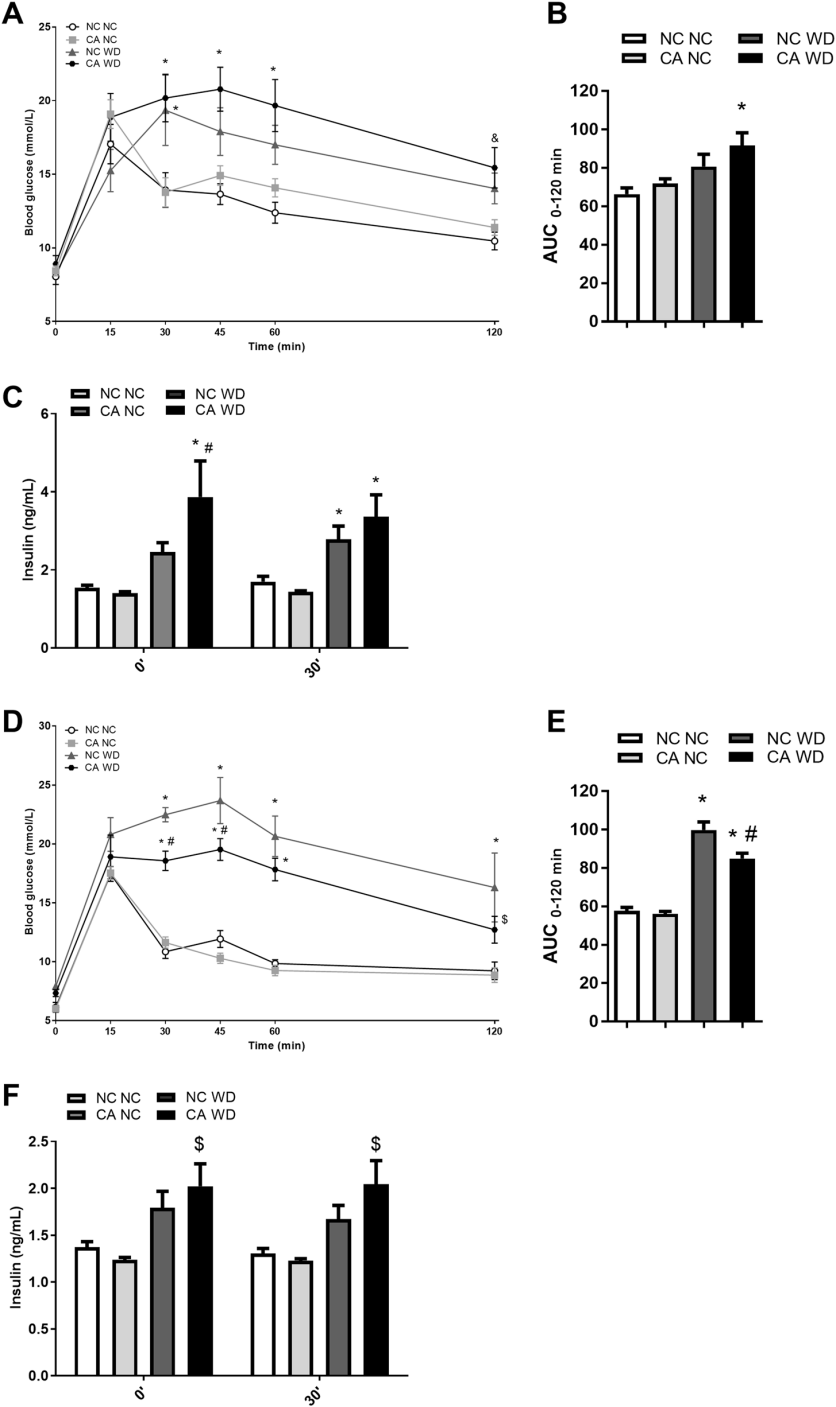
Glucose homeostasis in offspring. **a**, **b** Glucose tolerance test (GTT) and area under the curve (AUC) in male offspring; *n* = 6–8. **c** Measurement of insulin levels in male offspring prior to and 30 min after glucose injection; *n* = 6–8. **d**, **e** GTT and AUC in female offspring; *n* = 4–8. **f** Measurement of insulin levels in female offspring prior to and 30 min after glucose injection; *n* = 4–8. Data are presented as mean ± SEM. *P ≤ 0.05 for effects due to WD challenge in comparisons vs NC NC and CA NC; ^&^*P* ≤ 0.05 for effects due to WD challenge in comparisons vs NC NC; ^$^*P* ≤ 0.05 for effects due to WD challenge in comparisons vs CA WD; ^#^*P* ≤ 0.05 for effects due to paternal cholestasis in comparisons vs NC WD. Repeated measures two-way ANOVA followed by Tukey's post hoc test was used on GTT data comparisons, repeated measures one-way ANOVA followed by Newman–Keuls post hoc test was used on AUC and insulin measurements data

A GTT was performed at 18 weeks of age, and circulating insulin was measured prior to glucose injection, and 30 min after glucose injection. Male CA WD offspring showed a trend towards higher blood glucose levels compared to matched NC WD offspring during the GTT (Fig. 3a, b). CA WD male offspring also had significantly raised fasting insulin levels compared to NC WD controls (3.9 ± 0.9 vs 2.5 ± 0.2 ng/mL, respectively) (Fig. 3c). At 30 min after the GTT, a trend for increased insulin levels was still observed. No differences were seen in CA NC compared to NC NC offspring.

In contrast to male offspring, female WD-fed offspring of CA fathers showed improved glucose tolerance compared to NC WD at 30 and 45 min of the GTT challenge (Fig. 3d). Consistent with the GTT results, the AUC was 14% lower in CA WD female offspring compared to NC WD females (Fig. 3e). At the measured time-points, serum insulin levels were not different between CA WD and NC WD females (Fig. 3f). No differences were observed in female offspring fed a NC diet.

Overall, male and female offspring of CA fathers showed distinct glucose homeostasis phenotypes. Specifically, male CA WD offspring have increased fasting insulin when exposed to WD, whereas female offspring seem to be protected, to some extent, against WD-induced impairments in glucose tolerance.

### Paternal cholestasis affects lipid homeostasis in response to western diet differently in male and female offspring

**Fig. 4:**
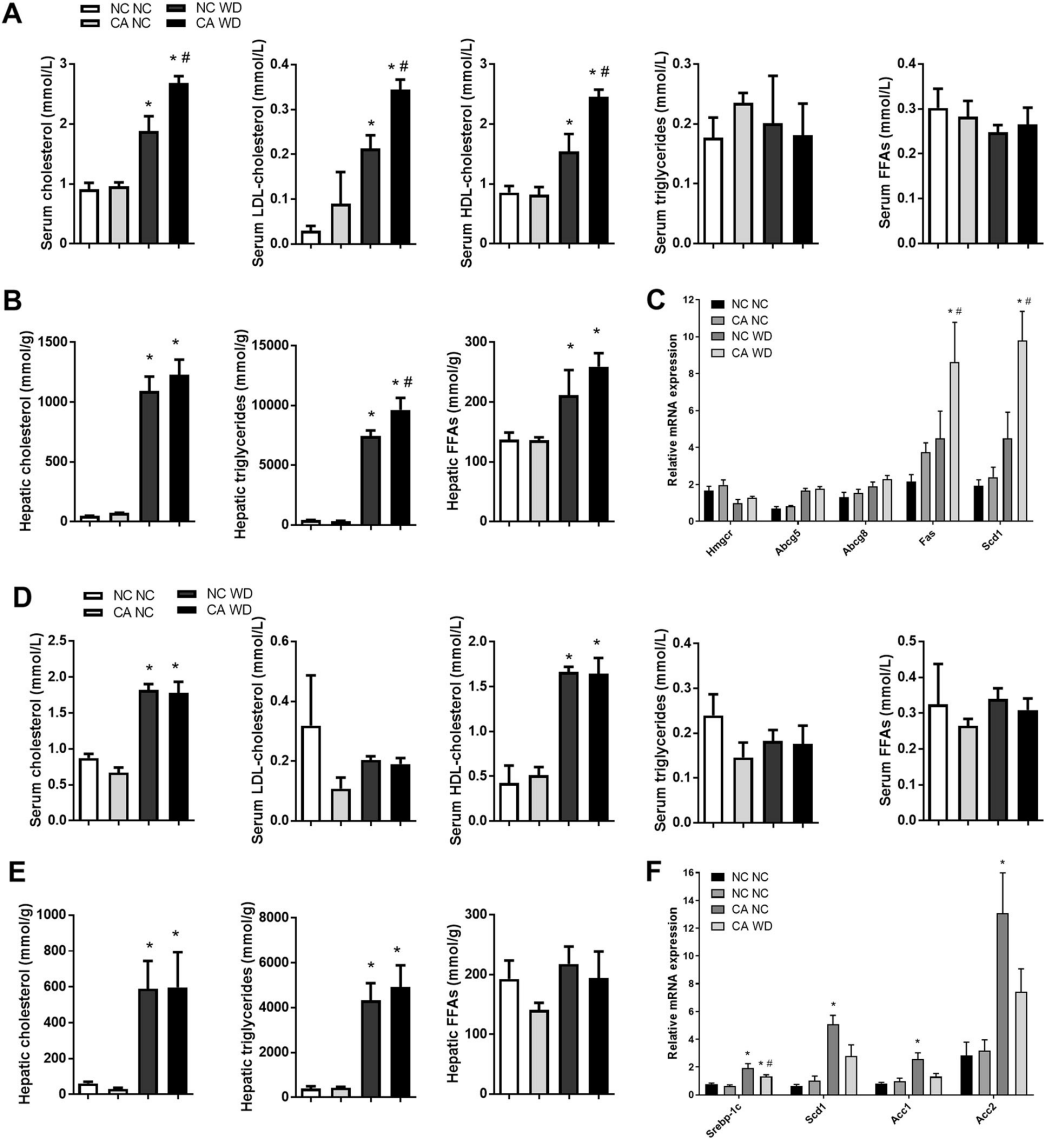
Serum and hepatic lipid profiles in offspring. **a** Serum lipid levels in male offspring; *n* = 6. **b** Hepatic lipid levels in male offspring; *n* = 6. **c** Hepatic expression of lipid homeostasis genes in male offspring; *n* = 6. **d** Serum lipid levels in female offspring; *n* = 4–6. **e** Hepatic lipid levels in female offspring; *n* = 4–6. **f** Hepatic expression of lipid homeostasis genes in female offspring; *n* = 4–6. Data are presented as mean ± SEM. *P ≤ 0.05 for effects due to WD challenge in comparisons vs NC NC and CA NC; ^#^*P* ≤ 0.05 for effects due to paternal cholestasis in comparisons vs NC WD. Repeated measures one-way ANOVA followed by Newman–Keuls post hoc test was used

We next investigated the serum and hepatic lipid profiles in the offspring of cholestatic fathers. WD-fed male offspring of cholestatic fathers had 43% higher levels of circulating total cholesterol, including a 62% increase in LDL-cholesterol and a 60% rise in HDL-cholesterol as compared to NC WD (Fig. 4a). However, the changes observed were not attributable to an increase in hepatic gene expression of *Hmgcr*, the rate-limiting enzyme for cholesterol synthesis, or the cholesterol exporters from the liver *Abcg5* and *Abcg8* (Fig. 4c). In addition to raised circulating cholesterol, CA WD males had a 30% increase in hepatic triglyceride content compared to NC WD controls (Fig. 4b). These findings were consistent with an increase in the hepatic expression of enzymes involved in key steps of fatty acid synthesis, i.e., *Fas* and *Scd1* that were raised by 0.9- and 1.2-fold respectively (Fig. 4c). Enhanced synthesis and storage of FFAs as TGs are likely to contribute to the increased hepatic triglyceride content in CA WD progeny. No changes in serum or hepatic lipids were seen in NC-fed male offspring.

In female CA WD and CA NC offspring, no changes in serum or hepatic lipid levels were found when compared to controls fed a matched diet (Fig. 4d, e). However, a decrease in the hepatic gene expression of *Srebp-1c*, *Scd1*, *Acc1* and *Acc2*, involved in controlling fatty acid biosynthesis, was seen in CA WD females as compared to NC WD controls (Fig. 4f).

Our results demonstrate that while male CA WD progeny have increased lipid accumulation in the liver and circulation, female offspring do not show a worsened lipid metabolic phenotype as a result of WD.

### Paternal cholestasis increases the risk of obesity-associated hypertension in male offspring and paternal UDCA treatment prevents elevated in blood pressure

Since CA WD male offspring showed several adiposity-associated cardiovascular risk factors, we next interrogated the effects of paternal cholestasis on the cardiovascular phenotype of the obese male offspring.

The phenotype of fathers fed a CA+UDCA diet was largely similar to that of CA-fed fathers except for higher serum lipids, and a further suppression in the expression of the bile acid synthesis genes *Cyp7a1* and *Cyp8b1* compared to NC controls (Supplementary Figure 4).

**Fig. 5:**
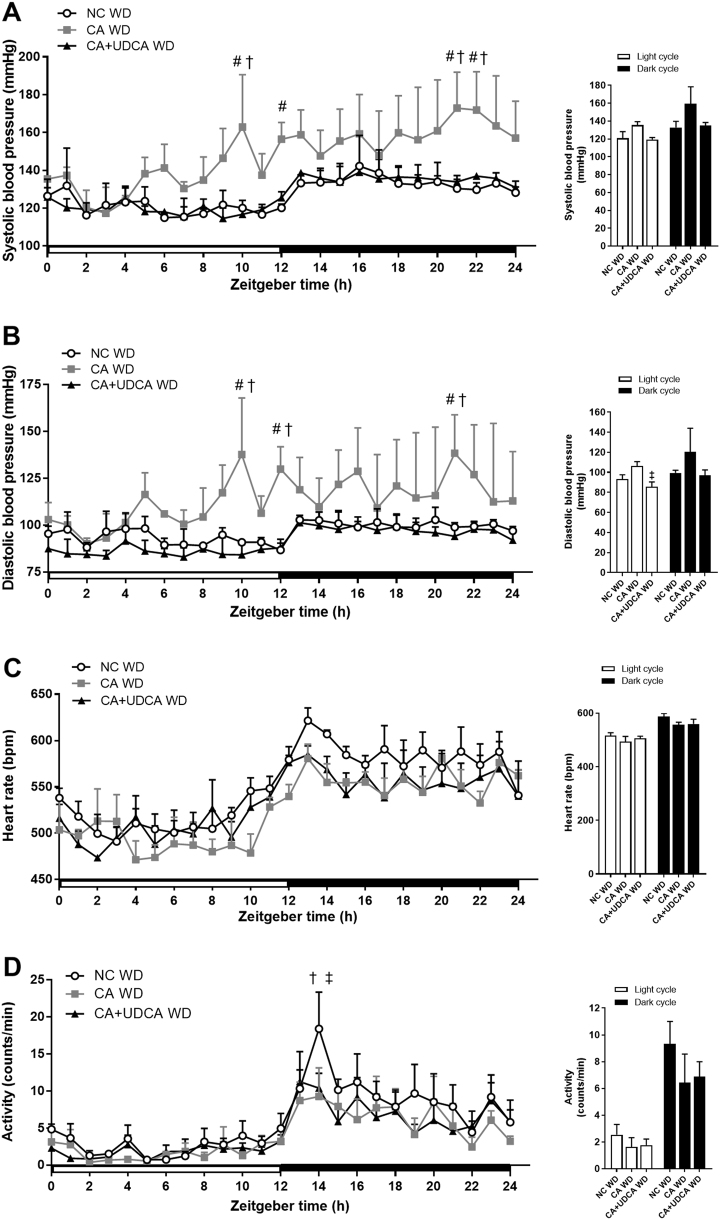
Effect of paternal cholestasis and ursodeoxycholic acid (UDCA) treatment of paternal cholestasis on cardiovascular parameters in the offspring. **a** Male offspring systolic blood pressure over a 24 h period and mean systolic blood pressure during the light and dark cycles. **b** Male offspring diastolic blood pressure over a 24 h period and mean diastolic blood pressure during the light and dark cycles. **c** Male offspring heart rate over a 24 h period and mean heart rate during the light and dark cycles. **d** Male offspring activity over a 24 h period and mean activity during the light and dark cycles. Data are presented as mean ± SEM; *n* = 3–4. ^#^*P* ≤ 0.05 for effects due to paternal cholestasis in comparisons vs NC WD; ^†^*P* ≤ 0.05 for effects due to untreated paternal cholestasis in comparisons vs CA+UDCA WD; ^‡^for effects due to paternal UDCA treatment in comparisons vs CA WD. Repeated measures two-way ANOVA followed by Tukey's post hoc test was used on 24 h recordings data comparisons, repeated measures one-way ANOVA followed by Newman–Keuls post hoc test was used on mean light and dark cycle comparisons

At 25–29 weeks of age, CA WD male offspring displayed a significant increase in systolic blood pressure at ZT 10, ZT 12, ZT 21 and ZT 22 (Fig. 5a). Significant elevations of diastolic blood pressure were also registered in CA WD males at ZT 10, ZT 12 and ZT 21 compared to NC WD controls (Fig. 5b). In contrast, CA+UDCA WD males had blood pressure levels comparable to those observed in NC WD controls. In particular, at ZT 10, ZT 21 and ZT 22 systolic blood pressure of CA+UDCA WD males was significantly lower than CA WD counterparts and comparable to NC WD controls (Fig. 5a). Diastolic blood pressure was also lower in CA+UDCA WD male offspring than CA WD males at ZT 10, ZT 12 and ZT 21 and did not differ from NC WD controls (Fig. 5b). There were no significant differences in heart rate between progeny groups (Fig. 5c), although NC WD male offspring were significantly more active than CA WD and CA+UDCA WD males at ZT 14 (Fig. 5d).

Despite the cardiovascular observations, no significant changes were seen in these older mice in body weight, liver weight or lipid profiles in CA WD and CA+UDCA WD males as compared to NC WD controls, although there was a trend for increased liver weight (Supplementary Figure 5).

These data indicate that paternal cholestasis is linked to increased predisposition of male offspring to developing obesity-associated hypertension, whereas paternal UDCA treatment of cholestasis can protect against hypertension in the offspring.

## Discussion

This study has shown that paternal cholestasis is linked to increased susceptibility to adiposity-associated metabolic and cardiovascular disease in the male offspring, and that paternal UDCA treatment can prevent the development of obesity-associated hypertension in the male offspring.

Offspring of cholestatic fathers did not show a disease-prone phenotype compared to controls until challenged with an obesogenic diet, which differentially affected male and female offspring and resulted in contrasting metabolic phenotypes at 18 weeks of age. When fed the same calorie-rich diet as controls, male offspring of cholestatic fathers showed features associated with the initial stages of metabolic disease, including higher fasting insulin levels and enlarged livers with increased hepatic triglyceride content. We also observed an increase in hepatic *Fas* and *Scd1* expression, markers of lipogenesis. Raised insulin levels and enhanced lipogenesis with hepatic triglyceride formation have previously been associated with the development of non-alcoholic fatty liver disease, where hepatocytes become insulin resistant and fail to repress lipogenesis and triglyceride accumulation in response to high insulin levels [23, 24]. Moreover, CA WD males showed a decrease in sWAT mass concomitant with hepatic TG accumulation that could be due to impaired sWAT expansion, as TGs have been shown to accumulate in the liver and muscle in the absence of functional adipocytes [25]. Males fed a WD also had higher serum cholesterol levels, but with no apparent increase in hepatic cholesterol biosynthesis or export.

Given the cardiovascular risk factors observed in the CA WD offspring, we used radiotelemetry to investigate the cardiovascular phenotype of older obese male offspring of cholestatic fathers, after being exposed to a WD for over 13 weeks. The male CA WD offspring showed an elevation of both systolic and diastolic blood pressure. However, in offspring of cholestatic fathers treated with UDCA, systolic and diastolic blood pressure levels were comparable to NC WD controls. These data show that treatment of cholestatic fathers with UDCA was effective in preventing the development of obesity-associated hypertension in male offspring beyond the levels registered in control NC WD offspring. No increase in heart rate was observed in male CA WD and CA+UDCA WD offspring compared to controls, suggesting that the baroreceptor reflex remained sensitive to raises in blood pressure.

The mechanisms underlying the increased adiposity-associated cardiometabolic disease susceptibility observed in the male offspring of cholestatic fathers and the protective effect given by paternal UDCA treatment against diet-induced hypertension are unknown but may involve persistent alterations in the paternal sperm epigenome, as observed in previous studies [7–10, 12, 15, 16, 26, 27]. Future studies will address potential changes in the sperm epigenome of cholestatic fathers and cholestatic fathers treated with UDCA that may affect the embryonic developmental trajectory and alter the phenotype of the adult offspring. In addition, investigation of the effects of paternal UDCA administration alone will be pursued to elucidate the protective effect of UDCA treatment of paternal cholestasis against obesity-associated hypertension in the offspring.

In contrast to male offspring, CA WD female offspring showed improved glucose tolerance compared to matched controls, in parallel with a decrease in the hepatic expression of the lipogenic genes *Srebp-1c*, *Scd1*, *Acc1* and *Acc2*. Overall, these data suggest that protective mechanisms may be in place in female offspring of cholestatic fathers to counteract the negative effects of exposure to WD feeding. Discrepancies between male and female offspring phenotypes have previously been reported in rodent models of paternal exposure to low-protein diet and obesity [10, 11, 28]. However, the mechanisms underlying the sexual dimorphism found in these models have not been described. Sex-specific effects have also been observed in models of fetal exposure to an adverse intrauterine environment. For example, in a rodent model of placental insufficiency, sex-specific effects on the cardiovascular phenotype of the offspring have been linked to reproductive hormones including estrogen and testosterone [29]. Moreover, sex chromosomes and genomic imprinted regions may play a role in influencing the developmental trajectory of the offspring. For example, in murine embryonic stem cells, sex chromosome complement alone has been associated with DNA methylome-wide differences [30] and with differential autosomal expression of coding and non-coding RNA [31]. In addition, differential expression of imprinted loci has been observed in male and female murine embryonic stem cells, despite maintaining correct parental imprinting patterns [31] and studies performed on the liver of adult mice have shown sex-dependent genome imprinting effects that were correlated to complex traits such as body and liver weight [32]. A further study has identified 1184 differentially methylated CpG sites between men and women, enriched at imprinted genes and distributed across all autosomes [33]. It is thus possible that changes to the paternal epigenome may interact with different factors present in the early embryo and later in life, including the sex chromosomes, genomic imprinted regions and reproductive hormones, to differently modulate the embryonic developmental trajectory and adult metabolic phenotype of male and female offspring from cholestatic fathers.

A limitation of the study is that during the mating period the paternal CA diet was discontinued and thus circulating bile acid levels would have gradually decreased in the CA group over the mating period. This would explain less marked changes in hepatic *Cyp7a1* and *Bsep* expression than would be anticipated. It was necessary to discontinue the paternal CA diet during the mating period to avoid exposing females to CA, which has previously been shown to influence the offspring phenotype later in life [20]. However, mean time to mate was approximately 3 days in the CA group and fathers would still have a significant derangement in bile acid and lipid levels caused by 10 weeks of CA feeding at the time of mating. Another possible caveat of the study is that implantation of the radiotelemetry probe has previously been shown to result in weight loss in mice for up to 15 days [34] and may have influenced the metabolic phenotype of the male offspring.

It is also important to note that CA feeding of fathers resulted in a lower paternal body weight despite causing hepatomegaly and dyslipidemia. Previous models of bile acid feeding have reported similar findings with liver enlargement secondary to increased hepatocyte size, dilation of interlobular bile ducts, parenchymal mitosis and inflammation in the liver [35, 36]. Dyslipidemia is also a feature of cholestasis since repression of *Cyp7a1* expression, an enzyme which catalyzes bile acid synthesis from cholesterol, results in hepatic cholesterol accumulation [37]. The observation that CA-fed fathers had smaller white adipose depots (gWAT and sWAT) was consistent with the decreased body weight in fathers throughout the CA feeding period. Rather than losing weight, CA-fed fathers appeared unable to gain weight at the same rate as controls, findings which align with a previous report of lower body weight in mice exposed to CA feeding for 102 days [38].

In conclusion, paternal cholestasis is associated with sex-specific effects on offspring susceptibility to metabolic disease and male progeny present a more disease-prone phenotype with features of metabolic disease and hypertension when exposed to an obesogenic diet. Paternal UDCA treatment has a protective effect against the exacerbation of obesity-associated hypertension in male offspring. The most prevalent cholestatic diseases in humans are primary sclerosing cholangitis and primary biliary cholangitis and UDCA is a common treatment for these conditions [18]. The results presented in this study warrant investigations into the metabolic and cardiovascular phenotype of children of fathers with primary sclerosing cholangitis and primary biliary cholangitis, who may be taking UDCA at the time of conception of their child.

## Electronic supplementary material

Supplementary figures

Supplementary materials and methods

Supplementary tables
