# Supplementary material for: Paternal cholestasis exacerbates obesity-associated hypertension in male offspring but is prevented by paternal ursodeoxycholic acid treatment
Source: Int J Obes (Lond). 2018 May 24;43(2):319–30. doi: 10.1038/s41366-018-0095-0 (PMC6124644; doi:10.1038/s41366-018-0095-0)
Supplement: Supplementary file 1 — Supplementary figures [file 41366_2018_95_MOESM1_ESM.docx]

**Supplementary figures**


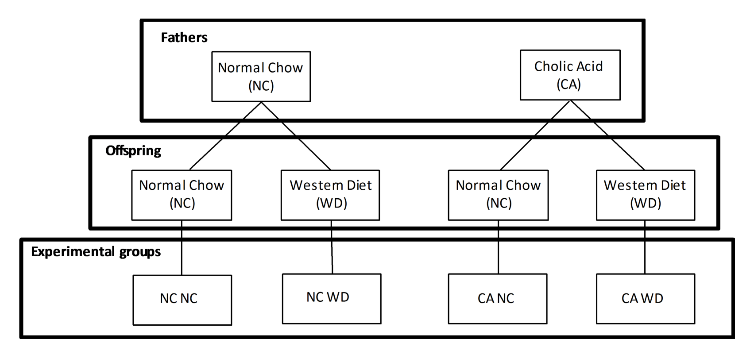


Supplementary Figure 1 – Breeding scheme used in animal experiments in cohort 1. Fathers were fed either a normal chow (NC) diet or a 0.5% cholic acid-supplemented (CA) diet. Offspring were fed a lifelong NC diet or challenged with a WD after 12 weeks of age. The combination of paternal and offspring diet exposures originated 4 offspring groups: NC NC, CA NC, NC WD and CA WD.


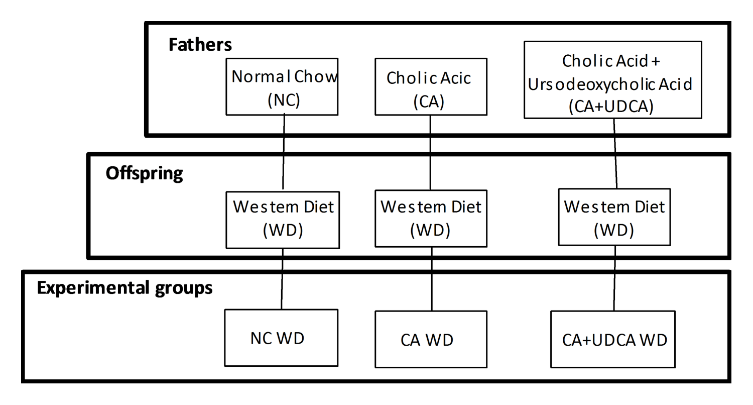


Supplementary Figure 2 - Breeding scheme used in animal experiments in cohort 2. Fathers were fed either a normal chow (NC) diet, a 0.5% cholic acid-supplemented (CA) diet or a 0.5% cholic acid + 0.5% ursodeoxycholic acid-supplemented (CA+UDCA) diet. Male offspring were challenged with a WD after 12 weeks of age. The combination of paternal and offspring diet exposures originated 3 offspring groups: NC WD, CA WD, and CA+UDCA WD.


Supplementary Figure 3 – Number of days between pairing of mating couples and the identification of a copulatory plug. n = 6 - 10. Data are presented as mean ± SEM. Unpaired 2-tailed t-test was used.

Supplementary Figure 4 - Paternal morphometry and metabolic profile in second cohort. (A) Paternal body weight during feeding period. n = 9 - 12. (B) Paternal organ weight. n = 9 - 12. (C) Paternal serum lipid levels. n = 7. (D) Paternal hepatic lipid levels. n = 7. (E) Paternal liver gene expression of bile acid homeostasis genes. n = 5 - 6. Data are presented as mean ± SEM. * P ≤ 0.05. Repeated measures of one-way ANOVA followed by a Newman-Keuls *post-hoc* test was used.


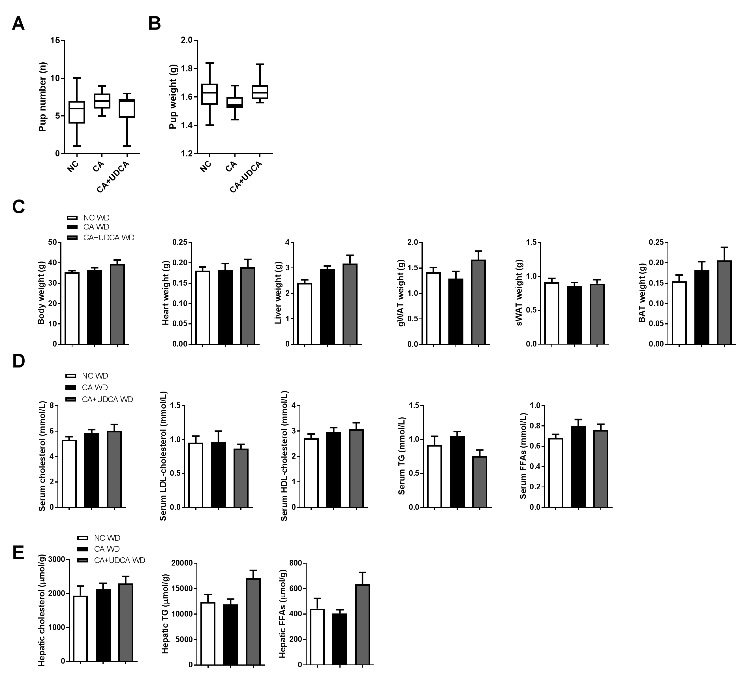


Supplementary Figure 5 – Offspring phenotype morphometry and lipid profile in second cohort. (A) Offspring birth weight. n = 9 - 12. (B) Offspring pup number. n = 9 – 12. (C) Male offspring body and organ weight at 25 - 29 weeks of age. n = 5. (D) Serum lipid levels in male offspring at 25 - 29 weeks of age. n = 5. (E) Hepatic lipid content in male offspring at 25 - 29 weeks of age. n = 5. Data are presented as mean ± SEM. Repeated measures of one-way ANOVA followed by a Newman-Keuls *post-hoc* test was used.
