# Supplementary material for: Paternal cholestasis exacerbates obesity-associated hypertension in male offspring but is prevented by paternal ursodeoxycholic acid treatment
Source: Int J Obes (Lond). 2018 May 24;43(2):319–30. doi: 10.1038/s41366-018-0095-0 (PMC6124644; doi:10.1038/s41366-018-0095-0)
Supplement: Supplementary file 2 — Supplementary materials and methods [file 41366_2018_95_MOESM2_ESM.docx]

**Supplementary materials and methods**

*Animal experiments*

All procedures were approved by the Animal Welfare and Ethical Review Body at King’s College London and carried out according to the UK Animals (Scientific Procedures) Act 1986. Male and female C57BL/6 mice aged 6-8 weeks were purchased from Harlan Laboratories, UK. Mice were housed on a 12 h: 12 h light: dark cycle with *ad libitum* access to food and water. Male mice were assigned to either a RM3 normal chow (NC) diet (n = 6) or a RM3 diet supplemented with 0.5% of cholic acid (CA) (n = 10) (LBS Serving Biotechnology, UK). Male mice were kept on the assigned diet for 10 weeks and body weight and food intake was measured weekly. Male mice fed a NC or CA diet were mated to NC-fed female mice of established fertility. Female mice used in the experiments had been previously mated and allowed to deliver 1 litter to establish fertility. During the mating period males were permanently kept with females and had access to *ad libitum* NC diet. Mating was confirmed by the presence of a copulatory plug, after which male mice were fasted for 4 h after 9 am and euthanized by CO2 inhalation. Serum, liver, gonadal white adipose tissue, subcutaneous white adipose tissue and testes were weighed and snap-frozen (NC: n = 6, CA: n = 10). Females were allowed to give birth and litters were left undisturbed for 24 h. On day 2, pup number per litter and pup weight were assessed and litters were standardized to 5-6 pups. In the NC group n = 6 litters were obtained, out of which 5 had both male and female offspring. In the CA group, n =10 litters were obtained, all of which had both male and female offspring. This influenced the number of biological replicates in downstream experiments. Offspring body weight was measured weekly. Pups were kept on a NC diet from 3 to 12 weeks of age, at which point half of the male and female offspring from each litter were challenged with a calorie-rich western diet (WD) (LBS Serving Biotechnology, UK) until 18 weeks of age. The combination of paternal and offspring exposure resulted in 4 experimental offspring groups: NC NC, CA NC, NC WD, and CA WD (first two letters: paternal dietary exposure; second two letters: offspring dietary exposure) (Supplementary Figure 1). At 18 weeks of age, one male offspring (NC NC: n = 6, CA NC: n = 8, NC WD: n= 6, CA WD: n= 8) and one female offspring (NC NC: n= 4, CA NC: n = 8, NC WD: n = 5, CA WD: n = 10) per litter were euthanized by CO_2_ inhalation after 4 h of fasting from 9 am and tissues harvested as described above. In a second cohort, the paternal feeding experimental protocol was repeated and a group of male mice fed a 0.5% cholic acid + 0.5% ursodeoxycholic acid (CA+UDCA)-supplemented diet (LBS Serving Biotechnology, UK) was included (NC: n = 10, CA: n = 12, CA+UDCA: n = 9). Male offspring were fed a NC diet until 12 weeks of age and then transferred to WD. The 3 offspring groups according to paternal and offspring diet were NC WD, CA WD, CA+UDCA WD (Supplementary Figure 2). Cardiovascular recordings were made in male offspring (25-29 weeks) by radiotelemetry (NC: n = 4, CA: n = 3, CA+UDCA: n = 4) (see below). In some assays, a randomly selected set of biological samples from each group was used rather than the full sample set.

*Radiotelemetry*

One male offspring per litter was anesthetized by isoflurane and surgical procedures were followed in a sterile environment. The probe catheter (Data Sciences International, USA) was implanted into the left carotid artery and the radiotelemetry transmitter unit then implanted subcutaneously on the left flank of the animal. Mice were single-housed and allowed to recover from surgery for 7 days before recording. Mice were then placed on telemetry receiver platforms and systolic blood pressure, diastolic blood pressure, heart rate and activity recorded for 3 days. Measurements for the last day of recording were plotted in hourly averages over a 24 h period and represented as zeitgeber time.

Following radiotelemetry, male offspring were fasted for 4 h and euthanized by CO2 and tissues were harvested.

*Lipid measurements*

Lipids were extracted from frozen liver samples using a lysis buffer containing 0.125M potassium phosphate, 1mM EDTA, 0.1% Triton X-100 (Sigma-Aldrich, UK) at pH 7. Serum and liver lipid extracts were run on a Unicel DxC 800 autoanalyzer (Beckman-Coulter, the Netherlands) for measurements of total cholesterol, LDL-cholesterol, HDL-cholesterol, triglycerides and free fatty acids. Liver lipid levels were subsequently normalized to protein content.

*Statistical analysis*

Group size of feeding groups was calculated to obtain sufficient statistical power based on previous similar publications. Animals were randomly assigned to a feeding group. Data are presented as mean ± SEM. Statistical analysis was performed using GraphPad Prism 7 software (GraphPad Software Inc., USA). Data was checked for normality using the Shapiro-Wilk normality test. For single comparisons unpaired 2-tailed t-test was used. F-test was used to test for equal variances between groups. Repeated measures of one-way ANOVA followed by a Newman-Keuls *post-hoc* test or two-way ANOVA followed by a Tukey *post-hoc* test were applied for multiple comparisons. Brown-Forsythe test and/or Bartlett's test were applied to test for equal variances between groups. The significance cut-off was *P* ≤ 0.05.
