# Supplementary material for: Paternal cholestasis exacerbates obesity-associated hypertension in male offspring but is prevented by paternal ursodeoxycholic acid treatment
Source: Int J Obes (Lond). 2018 May 24;43(2):319–30. doi: 10.1038/s41366-018-0095-0 (PMC6124644; doi:10.1038/s41366-018-0095-0)
Supplement: Supplementary file 3 — Supplementary tables [file 41366_2018_95_MOESM3_ESM.docx]

Supplementary tables

Supplementary Table 1 – Primer sequences.

| **Gene** | **Forward** | **Reverse** |
| --- | --- | --- |
| **Abcg5** | TCAATGAGTTTTACGGCCTGAA | GCACATCGGGTGATTTAGCA |
| **Abcg8** | TGCCCACCTTCCACATGTC | ATGAAGCCGCCAGTAAGGTAG |
| **Acc1** | GGCCAGTGCTATGCTGAGAT | AGGGTCAAGTGCTGCTCCA |
| **Acc2** | ACTTTGACCTGACCGCTGTG | CTGAGTGCCGGATATTGGC |
| **Bsep** | AAGCTACATCTGCCTTAGACAC | CAATACAGGTCCGACCCTCTCT |
| **Cyclophilin b** | TGGAGAGCCCAAGACAGACA | TGCCGGAGTCGACAATGAT |
| **Cyp7a1** | AGCAACTAAACAACCTGCCAGTACTA | GTCCGGATATTCAAGGATGCA |
| **Cyp8b1** | TAGCCCTCTTTCCTCCACTCAT | GAACCGATCGAACCTAAATTC |
| **Fas** | CCCAGAGGCTTGTGCTGACT | CGAATGTGCTTGGCTTGGT |
| **Hmgcr** | TTGGCACCATGTCAGGCGTCC | AGCGACACACAGGCCGGGAA |
| **Srebp-1c** | GCAGCCATGGATTGCACATT | GGCCCGGGAAGTCACTGT |
| **Shp** | CGATCCTCTTCAACCCAGATG | AGGGCTCCAAGACTTCACACA |
| **Scd1** | CCCCTGCGGATCTTCCTTAT | AGGGTCGGCGTGTGTTTCT |
